# Pediatric pulmonary nodules: current state of knowledge, AI applications, and future directions

**DOI:** 10.1007/s00247-025-06447-4

**Published:** 2025-11-12

**Authors:** Aki A. Tanimoto, Cara E. Morin, Andrew H. Schapiro, Eric J. Crotty, Andrew T. Trout, Jonathan R. Dillman, Russell C. Hardie

**Affiliations:** 1 Cincinnati Children’s Hospital Medical Center, Cincinnati, United States; 2 Department of Radiology, University of Cincinnati, Cincinnati, United States; 3 Department of Electrical and Computer Engineering, University of Dayton, Dayton, United States

**Keywords:** Children, Pulmonary nodules, Malignancy, Computed tomography, Artificial intelligence

## Abstract

Pulmonary nodules are commonly encountered in pediatric patients. While metastases make up the majority of malignant pulmonary nodules found in children, nodules are also frequently identified in healthy children. Currently, there are no definitive nodule features to differentiate benign from malignant nodules, and there are no guidelines for incidental nodule follow-up in children similar in scope to the 2017 Fleischner Society Guidelines for adults. In this review, we discuss the epidemiology, histology, and imaging findings of pulmonary nodules found in healthy children, pulmonary metastases, primary pediatric lung cancer, and other causes of pulmonary nodules in children. We also explore current applications of artificial intelligence for the evaluation of pediatric pulmonary nodules.

## Introduction

A pulmonary nodule is defined as “a circumscribed, typically round opacity, less than or equal to 30 mm in average diameter” by the Fleischner Society Glossary for Terms of Thoracic Imaging [1]. Nodules measuring greater than 30 mm are referred to as masses. Nodules measuring less than 6 mm in diameter are termed micronodules according to the 2024 Fleischner Society definition, a distinction used to distinguish potentially actionable (larger) nodules in adult patients [1]. Nodules can be described by their shape (round, ovoid, polygonal), margin (smooth vs. irregular), attenuation (solid, ground glass, part solid), and internal characteristics (fat, calcification, cavitation). They can also be classified according to their distribution – centrilobular, perilymphatic, or random. These characteristics can be used to predict the histopathology of nodules, and in adults, outcomes.

While pulmonary nodules are more commonly found in adults, they are also frequently encountered in both healthy children and those with known systemic disorders and malignancies, including extra thoracic and primary pulmonary malignancies. The 2015 Society for Pediatric Radiology Thoracic Imaging Committee publication, The Incidental Pulmonary Nodule in a Child: Part 2, provides a framework for the diagnostic management of nodules incidentally detected on CT [2]. However, it is important to note that there are currently no pediatric-specific guidelines for the management of pulmonary nodules similar in scope to the 2017 Fleischner Society guidelines for the management of incidental nodules found in adults who are at least 35 years old [3]. Therefore, those interpreting pediatric imaging studies must be knowledgeable about the etiology, prevalence, and imaging characteristics of pulmonary nodules in children to provide appropriate recommendations for follow-up evaluation if needed. Among these considerations, the presence or suspicion of underlying malignancy remains the most critical determinant influencing management decisions.

## Pulmonary nodules in healthy children

The widespread use of computed tomography (CT) has led to increased detection of pulmonary nodules in children, including incidental nodules. Incidental pulmonary nodules are nodules that are unexpectedly discovered on imaging exams, distinguishing them from those detected in patients with known malignancy or immunodeficiency disorder. Recently, ultra-high-resolution CT (U-HRCT) has become commercially available, offering superior spatial resolution through advanced detector technology, including energy-integrating and photon counting detectors, smaller detector element sizes, and large reconstruction matrices (1024 × 1024 and 2048 × 2048), which can be combined with deep learning-based algorithms for improved image quality [4–6]. In adult studies, U-HRCT has demonstrated improved image quality of pulmonary nodules compared to conventional CT and improved detection of nodules measuring less than 1 mm in diameter [4, 5]. As U-HRCT use expands in pediatric practice, radiologists are likely to identify more micronodules, underscoring the need for a clearer understanding of their prevalence and clinical significance in healthy children.

A small number of studies have evaluated the prevalence of pulmonary nodules in healthy children. Studies by Renne et al. and Samim et al. evaluated chest CTs of children who underwent imaging after trauma and found that 33–38% of patients had at least one nodule, with 1–5 nodules per patient [7, 8]. No children with malignancy were included in these studies. A study by Avles et al. evaluated the presence of pulmonary nodules in patients who underwent chest CT for preoperative workup of pectus deformity [9]. In this study, 75% of the children were found to have at least one pulmonary nodule, with up to 21 nodules per patient. The high frequency of pulmonary nodules in this study was thought to be secondary to endemic granulomatous disease. Exclusion criteria for this study included malignancy at the time of the exam or two years after, infection or trauma within 15 days before the scan, and any change in pulmonary or cardiac function tests during the preoperative evaluation.

Studies have also evaluated the clinical significance and implications of newly detected pulmonary nodules in both healthy children and children with systemic disease. A study by Breen et al. evaluated pulmonary nodules found on abdominal CT over a 7-year period and found that only 1.2% of patients had a newly detected pulmonary nodule. 81% (46/57) of nodules with follow up were found in patients without a history of malignancy, all of which were benign [10]. Notably, 5 mm slice thickness images were used for review, which likely limited the detection of micronodules. Additionally, Barber et al. found that 64% of children included in their study had a single nodule, 84% of whom were otherwise healthy [11]. Of the patients with multiple pulmonary nodules, 50% were otherwise healthy, while the remainder had systemic disease other than malignancy, known or diagnosed at the same time the nodules were found. Patients with malignancy and with chest CT performed for a positive tuberculosis screen were excluded from this study.

While few, these studies illustrate the frequency with which pulmonary nodules are found in children without malignancy, many of which are incidentally detected.

### Imaging features of benign nodules in children

**Fig. 1:**
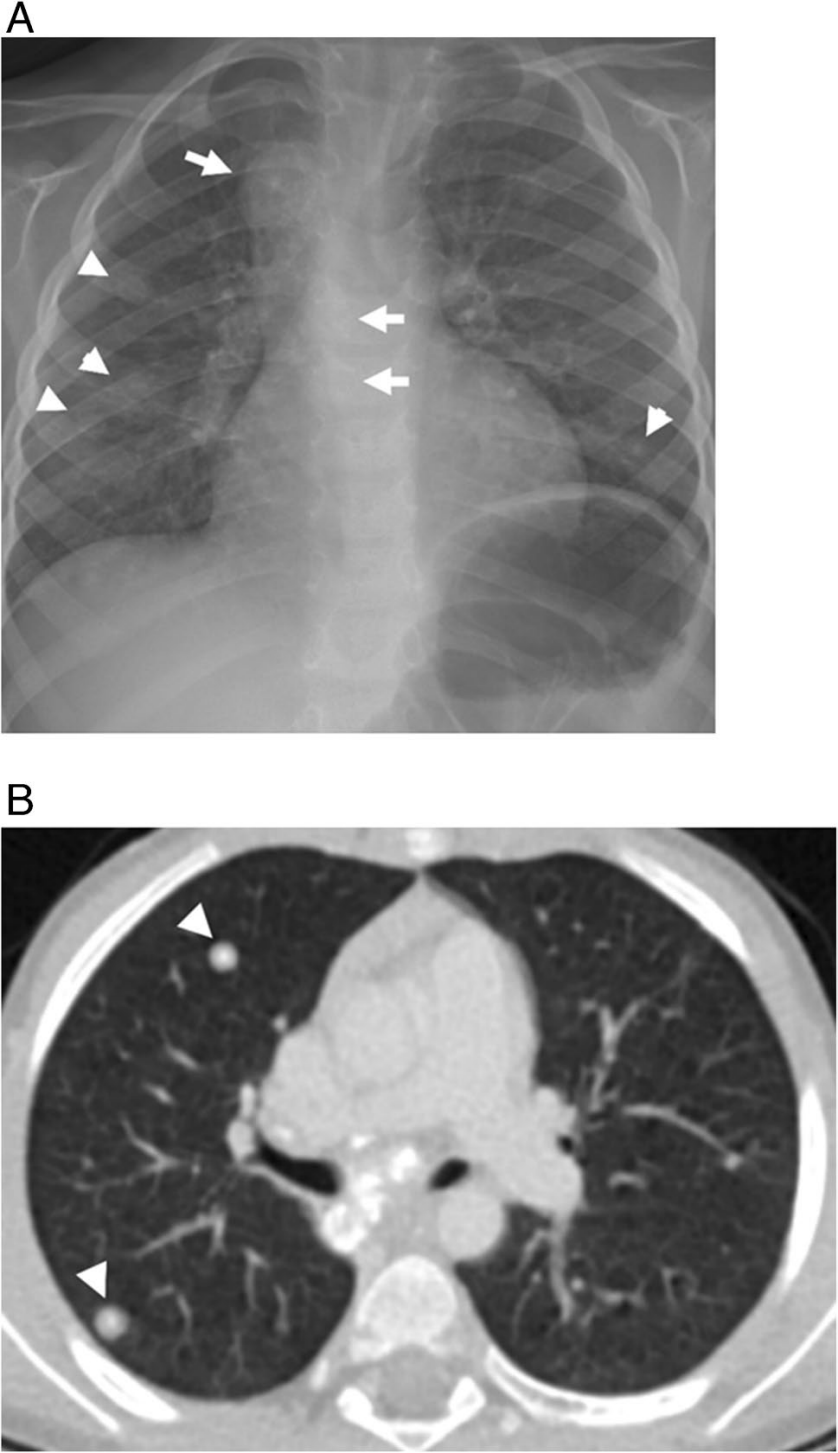
Sequela of prior granulomatous disease in a 6-year-old male with cough and fever. **A** Frontal chest radiograph shows enlarged, calcified mediastinal lymph nodes (*arrows*) and bilateral pulmonary nodules (*arrow heads*). **B** Contrast enhanced axial CT shows centrally calcified nodules (*arrow heads*). Findings are most consistent with sequelae of prior granulomatous infection, likely histoplasmosis given residency in the Ohio River Valley

**Fig. 2:**
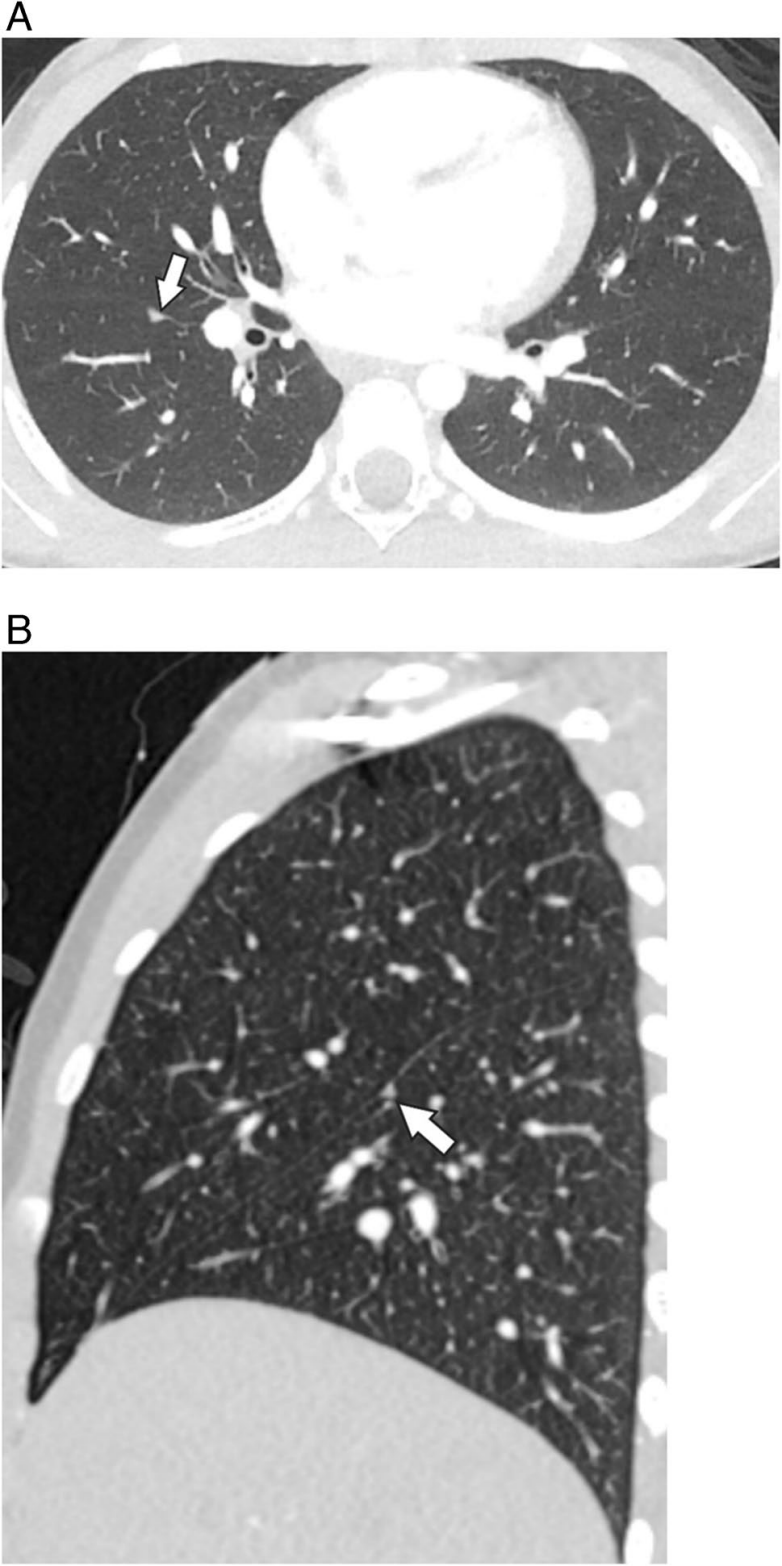
Benign perifissural lymph node in a 9-year-old female who presented with chest pain after gymnastics injury. **A** Axial and (**B**) sagittal contrast enhanced CT show an incidental 3 mm triangular, solid pulmonary nodule abutting the right major fissure that is most consistent with a lymph node (*arrows*)

Incidental pulmonary nodules are most commonly identified in the lower lobes [7–9]. They are typically less than one centimeter in size, with mean measurements around 3 mm (Table 1), and may contain calcifications (Fig. 1) [7–9]. CT features of benign intrapulmonary lymph nodes (IPLN), one of the several causes of benign incidental nodules in children, include triangular shape, size less than 1 cm (mean size 3.6 mm), smooth margins, and solid composition without internal calcification or fat (Fig. 2) [12]. A study of CT features of benign IPLNs in children with cancer found that benign IPLNs were frequently perifissural in location, similar to adult studies [12–14].

**Table 1:** Benign pulmonary nodule size

Study	Age	Patient characteristics	Avg nodule size
Renne	Mean, 9.4 years; Range 0–18 years	Chest CT in the emergency following trauma Excluded: children with known malignancy	2.6 mm (range 1–5 mm)
Samim	Median, 8 years; Range 1–12 years	Chest CT following high-energy trauma Excluded: children with history of malignancy	3.2 mm (2–6 mm)
Alves	Mean, 13.5 years; Range 4–18 years	Chest CT for preoperative assessment for pectus carinatum or excavatum Excluded: children with malignancy at time of exam or 2 years after, infection or trauma within 15 days before scan, any change in pulmonary or cardiac function tests during the preop evaluation	2.8 mm (2–8 mm)
Breen	Mean, 11.2; Range, 5 months – 18 years	All patients who had abdominal CT during a 7-year period with “nodule,” nodular,” or “mass” included in radiologic report in reference to lung bases Excluded: children with nodules that had been detected on a prior CT or those with concurrent chest CT at time of initial abdominal CT	Benign: 4.7 mm ± 3 mm Malignant: 11.5 ± 6.4 mm
Cho	Mean, 12 years; Range, 1–18.2 years	Pathology confirmed benign intrapulmonary lymph nodes from patients with known extrapulmonary solid malignancy	3.6 mm (1.3–7.8 mm)

### Histology of benign pulmonary nodules in children

**Fig. 3:**
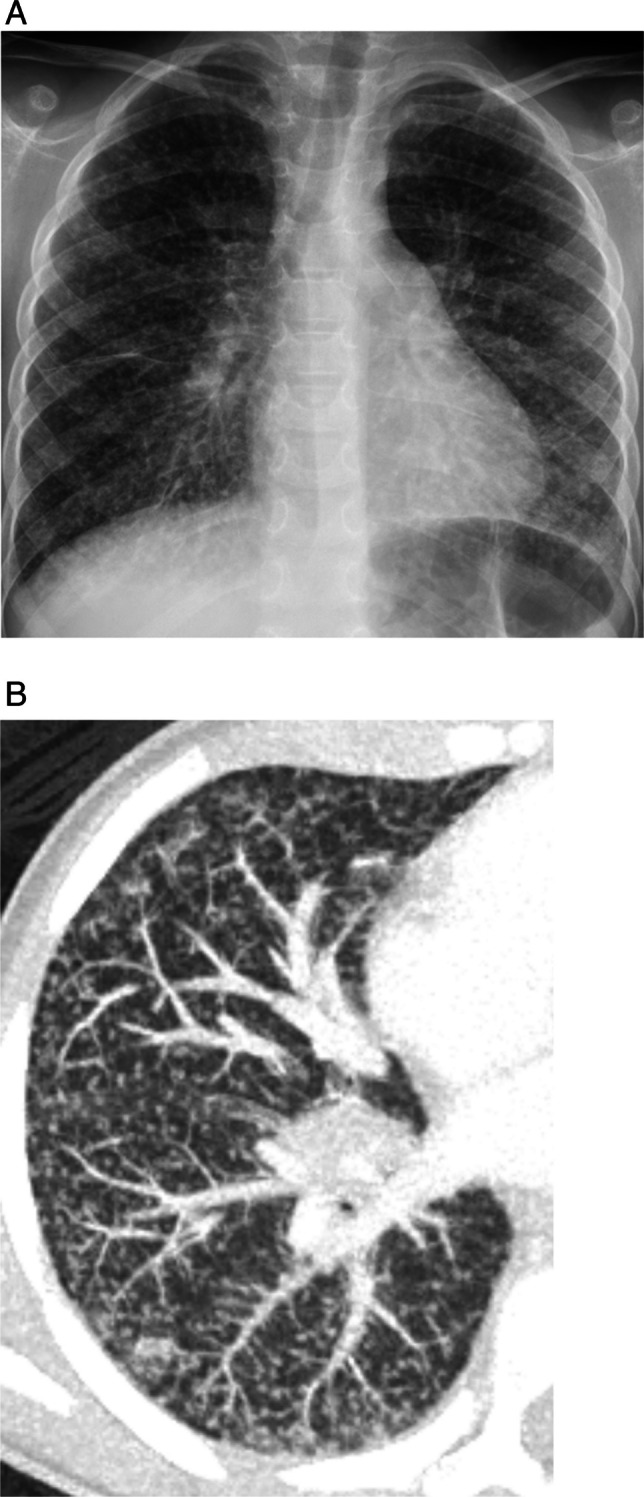
Acute disseminated histoplasmosis in a 6-year-old male presenting with worsening cough and fever. **A** Frontal chest radiograph demonstrates miliary nodules, (**B**) better visualized on CT with contrast enhanced axial MIP

In children without malignancy, pulmonary nodules are rarely biopsied and thus, the histology of these nodules is frequently unknown. By contrast, in children with extra-thoracic malignancies who undergo resection for suspected pulmonary metastases, the pathology of benign nodules has been characterized. Studies have reported the histopathology of resected benign nodules to include granulomas, fibrosis, lymph tissue, and infection amongst other causes (Fig. 3; Table 2) [15–18]. While unproven, benign nodules found in healthy children could have similar histopathology as those found in children with malignancy.

**Table 2:** Histopathology of biopsied nodules in children with known malignancy

Study	Age	Malignant	Benign	Benign nodule histology
Rosenfield	Median, 10 years; Range, 9 mo – 17 years	46% (6/13 biopsied nodules)	54% (7/13)	Inflammatory change, atelectasis, and lymph nodes
*McCarville	Median, 14.8 years; Range, 5–21 years	58% (24/41 patients biopsied)	42% (17/41)	Fibrosis, granuloma, lymphatic structure, hemorrhage, normal lung, lymphocytic infiltrate, infarct, dystrophic calcification, foreign body, papillary adenoma, and bronchopneumonia
Absalon	Median, 13.1 years; Range, 5.9–21.5 years	41.7% (10/24 patients biopsied)	58% (14/24)	Granulomas, normal lung tissue, interstitial fibrosis, or lymph tissue
**Silva	Median, 11.7 years; Range, 14 days – 17.8 years	33% (9/27 biopsied patients/nodules)	63% (17/27)	Not reported
Murrell	Median, 13 years; Range, 9 mo – 22 years	67% (44/68 biopsied nodules)	33% (23/68)	Bronchiolitis obliterans, granulomatous disease, atelectasis and pneumonia

*McCarville: 10% (4/41) both benign and malignant nodules on biopsy

**Silva: 4% (1/27) inconclusive biopsy

### Follow-up recommendations

Limited research on the natural history of incidental pulmonary nodules in children suggests that nodules without suspicious features detected in otherwise healthy children require no additional laboratory workup for infection and no repeat imaging [10, 11, 19]. Children with nodules associated with other systemic disorders typically present with other signs of the systemic disease [11]. The conclusion of these studies was echoed by the 2015 Society for Pediatric Radiology Thoracic Imaging Committee, which suggested that follow-up CT of incidental nodules may be of greater harm to children than benefit given the rarity of primary lung cancer in children, and suggested that management should be individualized based on clinical history and caregiver preference rather than following the adult-focused 2017 Fleischner Society Guidelines [20]. Based on this framework and the literature reviewed above, we propose that incidental nodules measuring ≤ 6 mm are likely benign, and in the absence of malignancy, do not require imaging follow-up. This aligns with the 2017 Fleischner Society guidelines for adults, making this size easy to remember, and is similar to the pediatric-specific recommendations proposed by Liang et al. in 2022, who suggested a slightly more conservative cutoff of 5 mm [21].

## Pulmonary metastasis in children

The vast majority of malignant pulmonary nodules represent metastatic disease from extrathoracic malignancies. Pediatric tumors that commonly metastasize to the lungs include Wilms tumor, osteosarcoma, rhabdomyosarcoma, Ewing sarcoma, hepatoblastoma, and, less commonly, neuroblastoma [20, 22–24]. While pulmonary nodules detected in children with known malignancy must be viewed with suspicion, they do not universally represent metastatic disease. A small number of studies have investigated the histology of biopsied nodules in children with known extra-thoracic malignancy (Table 2). For example, a study by Silva et al. found that 63% (17/27) of biopsied pulmonary nodules in patients with known extra-thoracic malignancy were benign, while 33% (9/27) were malignant; the remaining biopsy was inconclusive [25]. Similar findings were demonstrated in a study by Rosenfield et al. where 54% (7/13) of biopsied nodules demonstrated benign findings, whereas 46% (6/13) of biopsied nodules represented metastatic disease [17]. McCarville et al. found that 42% (17/41) of pediatric patients with malignant solid primary tumors who underwent biopsy had only benign nodules, 58% (24/41) had at least one biopsy-proven malignant nodule, and 10% (4/41) had both benign and malignant nodules [15]. These studies demonstrate how the indeterminate imaging features of pulmonary nodules in patients with known malignancy pose a challenge for radiologists and clinicians, as roughly half of the biopsied nodules in these studies were benign.

### Imaging features of metastatic nodules in children

**Fig. 4:**
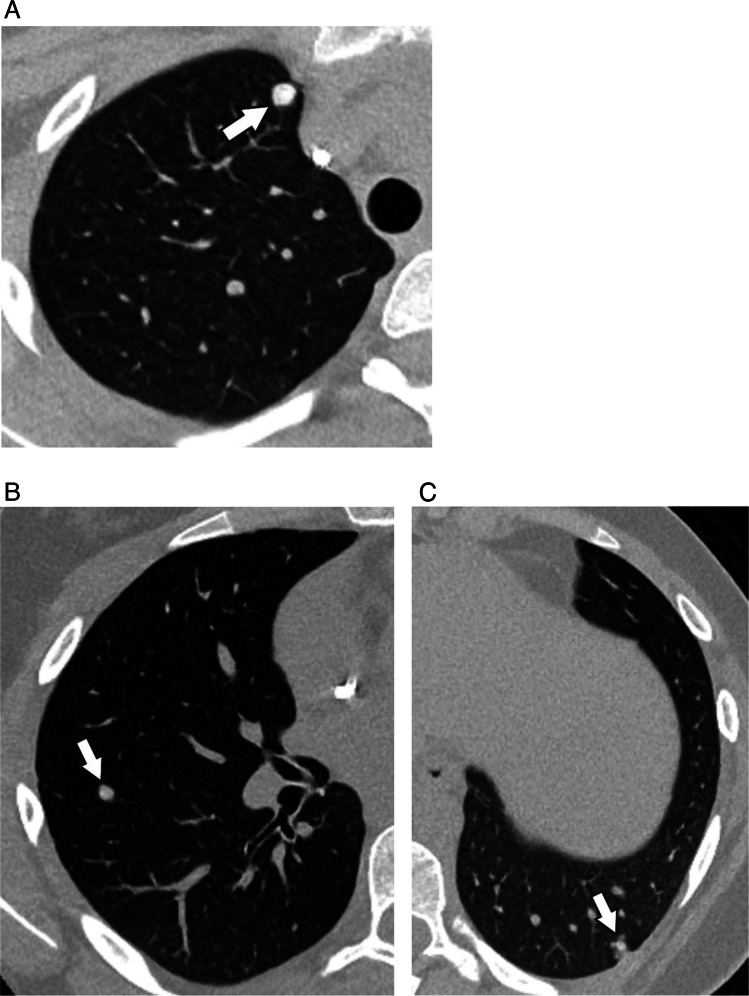
Metastatic osteosarcoma in a 13-year-old female. Non-contrast axial chest CT images (**A**-**C**) show multiple bilateral calcified nodules (*arrows*) compatible with metastasis, confirmed with surgical biopsy

**Fig. 5:**
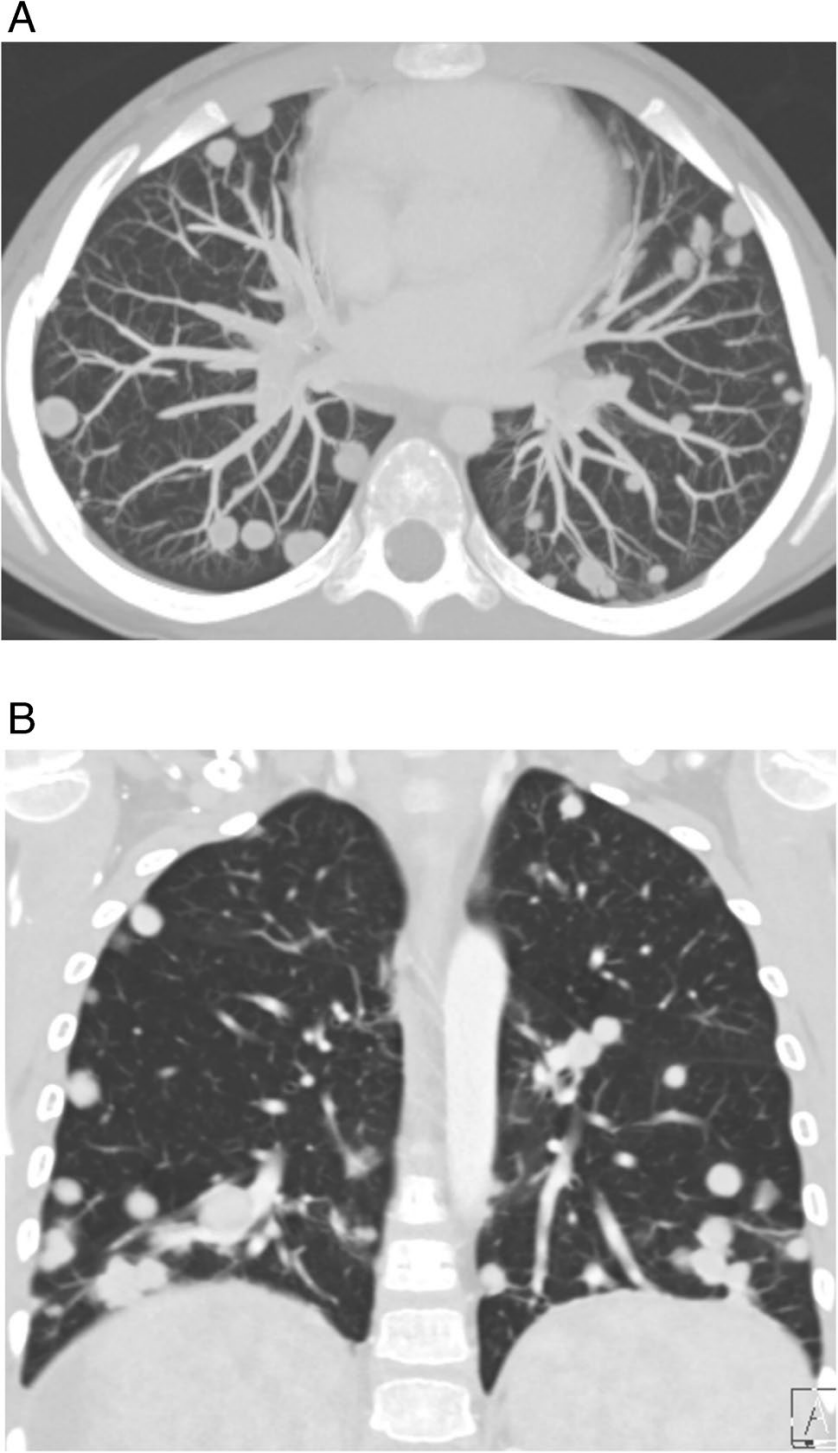
Metastatic hepatoblastoma in a 6-year-old male. Contrast enhanced (**A**) axial MIP and (**B**) coronal CT images show numerous, sharply marginated bilateral pulmonary nodules with a peripheral and lower lobe predominant distribution consistent with metastatic disease, confirmed with surgical biopsy

Given the relatively high prevalence of benign nodules in patients with known extra-thoracic malignancy, multiple studies have attempted to identify features distinctive of metastatic nodules. Perhaps the most suspicious feature of a metastatic nodule is the rate of growth. While rapid growth over the course of days is more often seen in infectious and inflammatory nodules, steady growth over the course of weeks should be viewed with suspicion. Likewise, nodules with sharply defined margins are more likely to represent metastases [15]. In patients with osteosarcoma, the presence of ossification/calcification is associated with an increased probability of malignant nodule histology (Fig. 4) [26, 27]. Nodule size, on the other hand, may or may not indicate malignancy. While some studies have found that nodules greater than 5 mm in diameter are more likely to be malignant, others have not found size to be associated with risk of malignancy [15–17, 26]. Thus, while larger nodules are more suspicious for malignancy, even tiny nodules should be viewed with suspicion in the child with known malignancy, as size alone is not a reliable discriminator between benign and malignant etiologies in children. Nodule number and distribution have also been studied in children with pulmonary metastases, with bilateral and peripheral distribution of nodules found to be associated with a higher likelihood of malignancy, consistent with hematogenous spread of metastatic disease (Fig. 5) [16, 18]. Thus, while CT imaging characteristics of pulmonary nodules can suggest metastasis, there are currently no definitive features of pulmonary metastases that distinguish them from benign nodules. Importantly, and in contrast to metastatic pulmonary disease in adults, the resection of pulmonary metastatic nodules in children with extra-thoracic solid tumors, particularly osteosarcoma, is a well-established practice and identification of all nodules can be important for surgical planning [28].

## Primary lung cancer in children

Primary lung malignancies in children are extremely rare, accounting for only 0.2% of all childhood malignancies, or approximately 1 in 2 million children [29]. The peak incidence occurs in children ages 0–4 years and 15–19 years [30].

A few studies have analyzed the National Cancer Data Base and the Surveillance, Epidemiology, and End Results (SEER) database to define the incidence of primary malignant pulmonary tumors in childhood and adolescence (Table 3) [29–31]. These analyses show that the most common primary pulmonary malignancy in children is carcinoid tumor, which is most commonly found in older children and adolescents [22, 29–32]. Other primary pulmonary malignancies include pleuropulmonary blastoma, most common in infants and young children, and mucoepidermoid carcinoma, typically presenting in older children and adolescents [33]. Squamous cell carcinoma accounts for a small portion of primary pediatric lung cancers, with an important risk factor in children being recurrent respiratory papillomatosis from human papillomavirus (HPV) infection (discussed below) [34].

**Table 3:** Primary pediatric lung cancer

2009 Analysis of the SEER database from 1973–2004	2015 Analysis of the National Cancer Data Base from 1998–2011	2023 Analysis of the SEER database from 2000–2019
Neuroendocrine tumors (n = 80, 51.6%) Sarcoma (n = 17, 11%) Mucoepidermoid (n = 14, 9%) Adenocarcinoma (n = 10, 6.5%) Pleuropulmonary blastoma (n = 7, 4.5%) Pulmonary blastoma (n = 7, 4.5%) Squamous cell carcinoma (n = 7, 4.5%) Small cell carcinoma (n = 7, 4.5%)	Carcinoid tumors (n = 133, 63%) Mucoepidermoid carcinoma (n = 37,18%) Squamous cell carcinoma (n = 19, 9%) Adenocarcinoma (n = 16, 8%) Bronchoalveolar carcinoma (n = 4, 2%) Small cell carcinoma (n = 2, < 1%)	Carcinoid tumors (n = 89, 29.6%) Pleuropulmonary blastoma (n = 67, 22.3%) Mucoepidermoid carcinoma (n = 37, 12.3%) Adenocarcinoma (n = 31, 10.3%) Neuroendocrine tumors (n = 67, 5.7%) Squamous cell carcinoma (n = 12, 5.3%) Atypical carcinoma (n = 7, 2.3%)

### Imaging features of primary malignancy

**Fig. 6:**
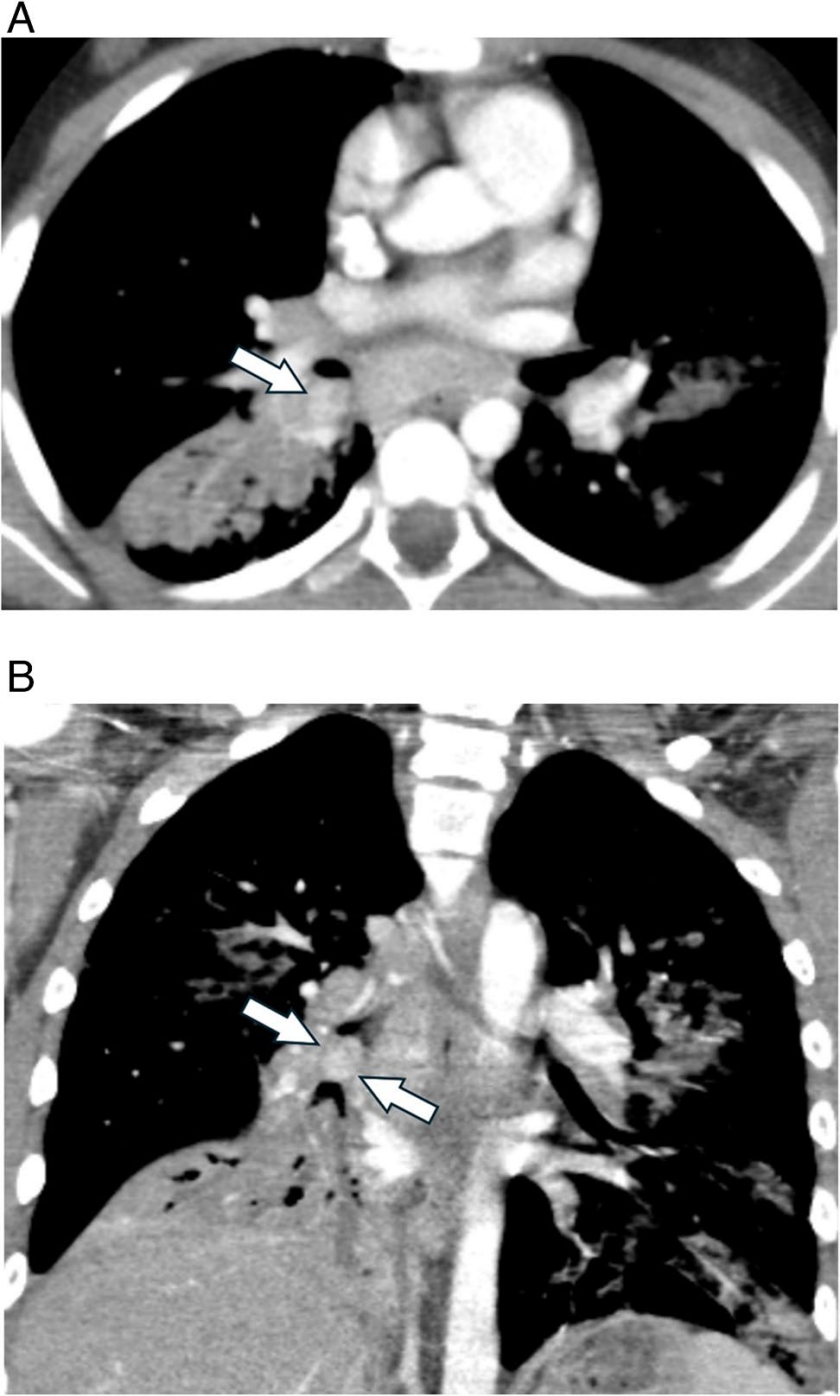
Carcinoid tumor in a 12-year-old female with chronic cough, fevers, and chest pain. **A** Axial contrast-enhanced chest CT shows an enhancing, endobronchial nodule at the junction between the right lower lobe bronchus and bronchus intermedius (*arrow*). **B** Coronal CT image shows airways distal to the endobronchial tumor (*arrows*) are mucus filled and dilated, and there is consolidation of the right lower lobe

While uncommon, primary malignant tumors may present as pulmonary nodules. For example, carcinoid tumors often present as a well-defined, hilar or perihilar, solitary nodule or mass [33, 35]. Carcinoid tumors may be completely endoluminal, partially endoluminal, or may abut the airway (Fig. 6) [35]. While most lesions are central, carcinoid tumors may also be found in the lung periphery [36]. Additional imaging features of carcinoid tumors include tumoral enhancement on contrast-enhanced CT and magnetic resonance imaging (MRI), and calcification (punctate, eccentric, or diffuse) [33, 35, 36]. Bronchial carcinoids typically demonstrate high signal intensity on T2-weighted and short time inversion recovery MR images [37]. Carcinoid tumors typically abundantly express the type 2 somatostatin receptor and will show robust uptake on ^68^ Ga-DOTATATE PET, which may be performed for tumor characterization, staging, and therapy planning.

**Fig. 7:**
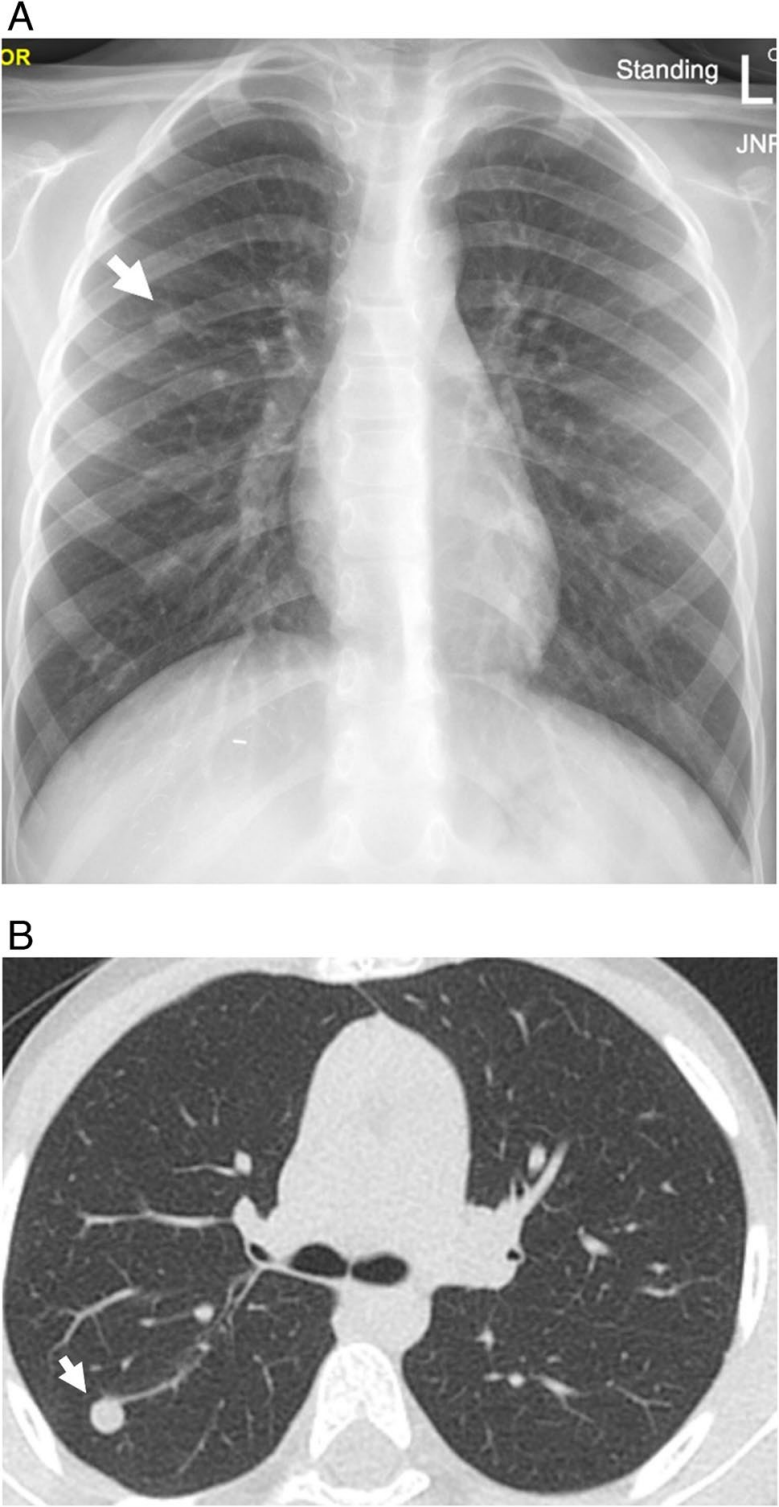
Mucoepidermoid carcinoma in a 6-year-old male with history of hepatoblastoma status post hepatic resection and chemotherapy 5 years prior. **A** Screening chest radiograph demonstrates a nodular opacity in the right upper lobe (*arrow*). **B** Non-contrast chest CT image shows a corresponding round, well defined right upper lobe nodule (*arrow*)

Mucoepidermoid tumors also present as well-circumscribed, endobronchial nodules or masses that may arise from central or peripheral airways (Fig. 7) [33, 38]. A study that included both pediatric and adult patients suggested that a “markedly enhanced central bronchial nodule or mass may suggest low-grade [tumor]. High-grade [tumor] tends to be peripheral, to have poorly defined margins, and to be lobular, heterogeneous nodules or masses with less enhancement” [39].

Other primary lung tumors, such as pleuropulmonary blastoma (PPB) and inflammatory myofibroblastic tumor (IMT), typically present as masses rather than nodules [33]. PPB is the most commonly seen malignant mass in neonates and infants. IMT is the most common primary mass of the lungs in children, and typically presents as a solitary, well-circumscribed, peripheral lung mass [33, 40]. While IMT is considered a benign tumor, its clinical behavior can be variable, with potential for recurrent and metastatic disease [41, 42]. Imaging features such as enhancement patterns and calcification are variable.

## Other causes of pulmonary nodules in children

### Infectious

Pulmonary nodules are commonly seen in the setting of infection and aspiration as clustered centrilobular nodules. In the setting of endemic fungal infection, particularly histoplasmosis and coccidioidomycosis, solitary, multiple scattered or miliary pattern nodules can be seen, with miliary pattern nodules typically seen in immunocompromised patients. Helpful clues for distinguishing a solitary nodule due to endemic fungal infection from lung malignancy or metastatic disease include peribronchovascular nodularity/beading adjacent to the nodule and/or tiny satellite nodules surrounding the dominant nodule. Nodules due to histoplasmosis tend to centrally or diffusely calcify over time and are often seen in the setting of calcified hilar lymph nodes [43]. Notably, given the wide spectrum of radiographic presentations of tuberculosis, high clinical suspicion is recommended in areas with endemic disease.

### Non-infectious

Pulmonary nodules in children may arise from a variety of non-infectious etiologies. These may occur in the context of various systemic conditions, including immunodeficiency/dysregulation, lymphoma, and post-transplant lymphoproliferative disorder [44, 45], connective tissue disorders [44], sarcoidosis [46] and vasculitides [47, 48]. For example, ill-defined centrilobular nodules have been described in patients with lymphoid interstitial pneumonia [44]. In granulomatosis with polyangiitis, the most common lung manifestations include multifocal nodules, many exceeding 5 mm in diameter, sometimes with cavitation in large nodules/masses [47].

While pulmonary hamartomas are commonly seen in adults, they are rare in children. Imaging features of hamartomas include internal fat and popcorn calcification in a well-circumscribed nodule with lobulated margins [49, 50].

*Pulmonary Langerhans cell histiocytosis* (PLCH), though uncommon, can lead to the formation of nodules. Unlike in adults, where most patients are cigarette smokers, the pathogenesis in children is thought to reflect an uncontrolled immune response to an unknown stimulus/antigen, and children more often present with the multifocal/systemic form of LCH. Similar to adults, nodules in children evolve and undergo cavitation, resulting in cysts with variable wall thickness and sizes, often bizarrely shaped [51].

**Fig. 8:**
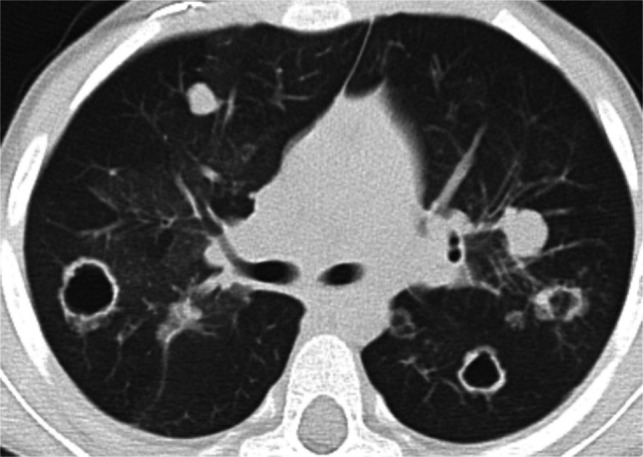
Recurrent respiratory papillomatosis in a 4-year-old male. Non-contrast axial chest CT shows bilateral solid and cavitary nodules

*Recurrent respiratory papillomatosis* (RRP) is a chronic disease caused by infection with HPV types 6 and 11, resulting in the growth of benign squamous papillomas in the aerodigestive tract. Juvenile-onset RRP is thought to occur through transmission from mother to infant during birth through contact with infected secretions in the birth canal. RRP is typically limited to the larynx and rarely involves the lungs. Pulmonary involvement of RRP is best evaluated with CT [52, 53]. Pulmonary lesions include scattered, well-defined, solid nodules, cavitary nodules, air-filled cysts, and masses predominantly in the lower and posterior lungs (Fig. 8) [33, 53, 54]. Signs concerning for malignant transformation of pulmonary RRP include “development of heterogenous mass with central necrosis in an area previously occupied by air-filled cysts, invasion of adjacent structures, pleural effusion, and mediastinal and cervical adenopathy” [33]. Additionally, ^18^F-fluorodeoxyglucose positron emission tomography (FDG PET) can be utilized to detect pulmonary lesions and mediastinal/hilar lymph nodes that are disproportionately avid [54, 55].

Additional causes of pulmonary nodules can be found in Table 4.

**Table 4:** Etiology of pulmonary nodules

Etiology	Type of pulmonary nodule
Infection	Granulomatous Bronchopneumonia Septic embolism Parasitic infection
Inflammation	Aspiration Vasculitis Sarcoidosis Hypersensitivity pneumonitis Granulomatous lymphocytic interstitial lung disease
Vascular lesion	Arteriovenous malformation Pulmonary infantile hemangiomas Plexiform lesions (pulmonary arterial hypertension)
Benign neoplasm	Papilloma/papillomatosis Lymphoproliferative disease Pulmonary Langerhans cell histiocytosis Chondroma Hamartoma
Other	Intrapulmonary lymph node Hemorrhage

## Imaging of pulmonary nodules

### Chest radiography

Chest radiography remains the most utilized imaging modality to evaluate thoracic disease in children. It offers a low-cost, readily available, and relatively fast and easy method of chest imaging. Additionally, exposure to ionizing radiation is very low with chest radiography. While chest radiographs are often obtained for the evaluation of pneumonia and other acute chest pathology, they are also commonly used for long-term surveillance for metastatic disease to the chest. In patients with favorable-histology Wilms tumor, surveillance for recurrent pulmonary metastatic disease has shifted from CT to radiography, as a study by Mullen et al. found that the elimination of surveillance CT scans was found to be unlikely to compromise survival, as there was no difference in the survival of patients with a maximum tumor diameter of less than 1 cm vs 1–2 cm at the time of recurrence [56]. Given the widespread use of radiography and the common presence of pulmonary nodules in both healthy children and those with malignancy, it remains important that those reading pediatric chest radiographs remain vigilant for pulmonary nodules, keeping in mind that many soft tissue nodules 1 cm or smaller are not visible on chest radiographs and that those visible that are smaller than 1 cm are often calcified or represent false positives [57].

### CT chest

**Fig. 9:**
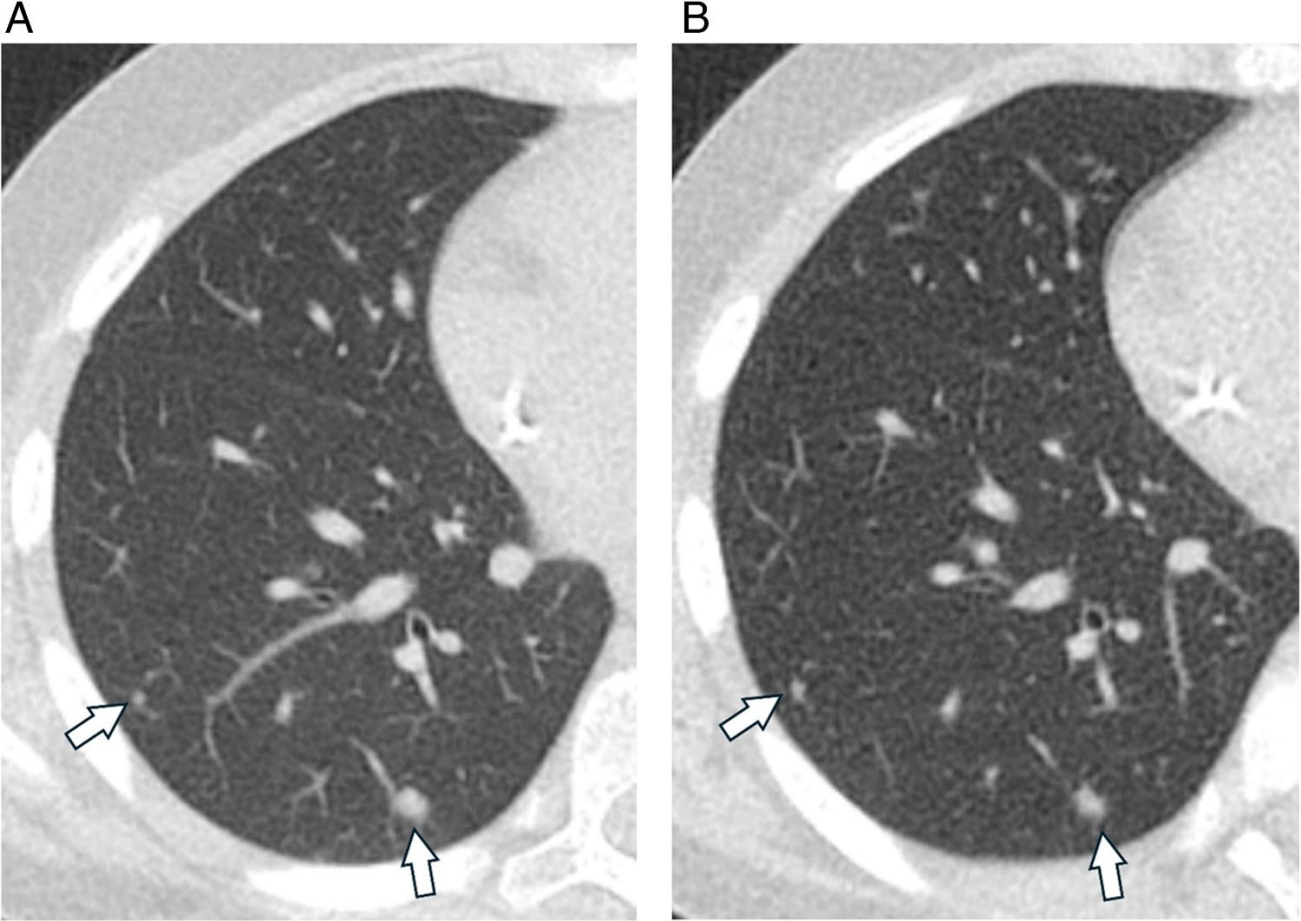
Clinical and reduced-dose CT images of an 11-year-old male with osteosarcoma. **A** clinical dose and **B** reduced dose axial CT images show two right lower lobe pulmonary nodules (*arrows*)

CT is the most used cross-sectional modality for the evaluation of known pulmonary nodules and is accepted as the reference standard for nodule detection. The use of multidetector CT allows for fast image acquisition, as well as high contrast and high spatial resolution imaging of the lungs. Rapid image acquisition is of particular benefit in children, as it may negate the need for sedation and its associated consequences, including complex scheduling with anesthesia, potential adverse effects of anesthetic agents, as well as anesthesia-associated atelectasis that limits the radiologist’s ability to detect pulmonary nodules [58, 59]. High spatial resolution (2 mm slice thickness and less) optimizes detection of pulmonary nodules, including micronodules. Additionally, the use of multiplanar reconstruction and maximum intensity projection (MIP) reformats has been shown to improve the detection of pulmonary nodules [60–62]. One study showed the addition of MIP images to increase detection of lung nodules from 57 to 71% in children under 13 years [60]. This is of particular importance in the evaluation for metastatic disease, as micronodules may represent pulmonary metastases and potentially alter staging and treatment course. While CT provides excellent evaluation of lung parenchyma, it traditionally exposes children to higher (vs. radiography) doses of ionizing radiation, and children face a higher lifetime risk of malignancy relative to adults [63–66]. As a result, dose reduction techniques have been leveraged to minimize radiation exposure to children who require CT imaging. For example, a study of pediatric pulmonary nodules by Thapaliya et al. achieved a 0.3 mSv mean effective dose with reduction of tube current to 40 or 25 mA, depending on the CT scanner used, at a tube voltage of 100 kVp [67]. Studies in children have demonstrated that pulmonary nodules can still be reliably detected with the use of dose reduction techniques (Fig. 9) [67–69]. Additionally, photon-counting detector CT (PCD CT) has been shown to significantly reduce radiation dose relative to energy-integrating detector CT (EID CT) in pediatric patients while maintaining objective and subjective image quality of chest CTs [70, 71]. Furthermore, studies evaluating image quality of adult nodules on PCD CT suggest improved image quality of nodules on PCD CT with greater spatial resolution compared to EID-CT [72]. Deep learning-based image reconstruction and restoration algorithms have also been developed for image noise reduction that allows low-dose CT image quality to be improved [73] (discussed below).

### MRI chest

MRI chest is most commonly used for soft tissue characterization of mediastinal and chest wall masses as it offers superior soft tissue characterization relative to CT. MR of the lung parenchyma is made difficult by the low proton density of aerated lungs, resulting in a low signal-to-noise ratio, susceptibility artifacts due to the tissue and air interfaces, as well as artifacts from respiratory and cardiac motion [74]. Despite these challenges, advances in MR technology, including ultrashort echo time and deep learning reconstruction of images, have resulted in the ability to detect pulmonary nodules as small as 5 mm without exposure to ionizing radiation under optimal scanning conditions [75–77]. Studies in adults have also demonstrated the potential for tissue characterization and differentiation of malignant from benign nodules measuring greater than 5 mm with diffusion weighted imaging and dynamic contrast enhanced imaging [78–81]. However, MR remains limited in its ability to reliably detect nodules smaller than 5 mm, thus limiting its current use as a tool for the detection of small pulmonary metastases [82, 83]. Another major limitation of MR is in the detection of calcified nodules, such as those seen in cases of metastatic osteosarcoma, as calcification results in susceptibility-related signal loss potentially rendering these nodules invisible on MR [76]. Other limitations of MR include longer image acquisition time and the need for patient cooperation, which is of particular concern when imaging children.

#### PET

In adults with solid pulmonary nodules > 8 mm and low to moderate pretest probability of malignancy (5%–65%), ^18^F-fluorodeoxyglucose positron emission tomography (FDG-PET) is recommended for further characterization [84]. While elevated FDG uptake is often identified in malignant nodules of sufficient size, it is not specific for malignancy and can also be seen in infectious/inflammatory nodules. In adults, FDG-PET/CT shows a sensitivity of 72%–94% for distinguishing malignant pulmonary nodules [84]. PET/CT is uncommonly performed to characterize indeterminate pulmonary nodules in healthy children without known malignancies, and there is no data on the accuracy of FDG-PET/CT in distinguishing benign from malignant pulmonary nodules in healthy children. McCarville et al. showed a sensitivity of 76% and specificity of 36% in detecting malignant pulmonary nodules ≥ 5 mm with FDG-PET/CT in children with solid tumors [85]. The sensitivity was unchanged, but specificity increased to 86% when reviewers evaluated a diagnostic chest CT in conjunction with the FDG-PET/CT, presumably reflecting the fact that PET imaging is typically performed free-breathing while diagnostic CT is performed with a breath hold or during suspended respiration. In the study by McCarville et al., no nodules < 5 mm were detected by FDG-PET/CT. The median maximum standard uptake values (SUVmax) of malignant nodules ≥ 5 mm were significantly higher (3.1) than those of benign nodules (1.1), and SUVmax was a significant predictor of malignant histology. PET technology has advanced since the study by McCarville et al. with increased scanner sensitivity and motion compensation techniques, which should increase detectability and improve characterization of metabolically active lung nodules. Like CT, PET scans necessitate ionizing radiation exposure to pediatric patients, and historically long image acquisition times require patient cooperation and, in some cases, necessitate patient sedation. Newer generation scanners with increased sensitivity and z-axis field of view have addressed some of these limitations, reducing patient doses and significantly shortening scan times.

## Artificial intelligence and pulmonary nodules

In recent years, the use of artificial intelligence (AI) in medical imaging has significantly increased due to decreased cost of powerful parallel computing hardware, including graphics processing units, increased availability of large quantities of labeled data as well as increasing use of synthetic data, and improvements in training techniques and machine learning architecture [86, 87]. The use of AI in chest imaging includes, but is not limited to, lung segmentation, detection of pulmonary nodules, pulmonary nodule characterization, and noise reduction techniques for low-dose CT [88, 89].

### Image acquisition and reconstruction

Advances in CT and PET/CT technology enabled by AI have allowed dose reduction for both adult and pediatric patients. While lowering dose has historically resulted in degradation of image quality due to increased noise in the reconstructed images, AI-based reconstruction algorithms have improved image quality in low-dose scans. For example, a study by Brady et al. evaluated a deep learning-based reconstruction (DLR) algorithm for CT that allowed for 52% greater dose reduction than statistical-based iterative reconstruction algorithms [90]. Moreover, DLR algorithms maintain spatial resolution and noise texture preferred by radiologists [73, 90, 91].

### Detection of pulmonary nodules

**Fig. 10:**
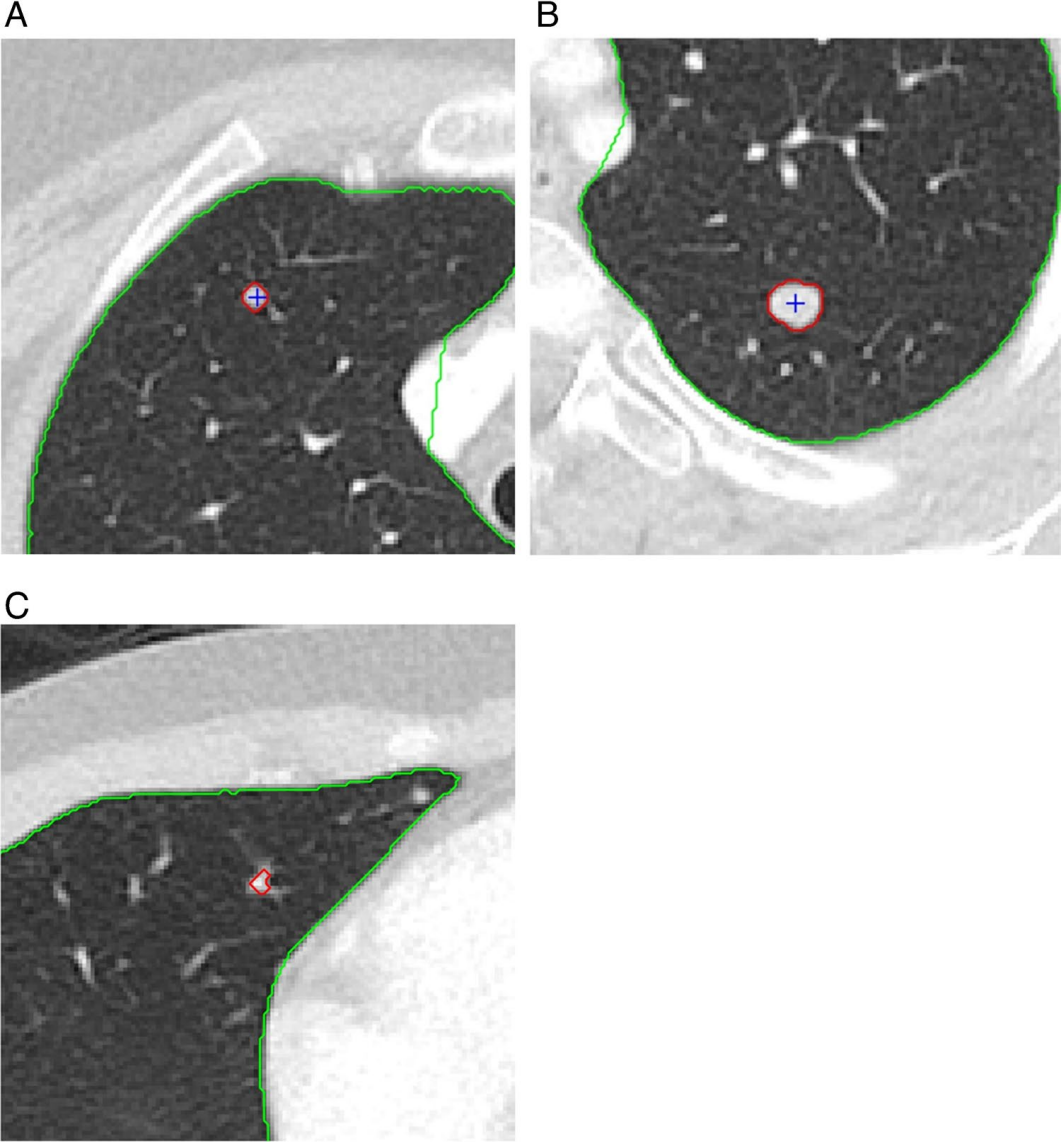
12-year-old M with hepatocellular carcinoma. CAD detected pulmonary nodules in the (**A**) right upper lobe and (**B**) left lower lobe. **C** False positive CAD system detection triggered by pulmonary vasculature in the right middle lobe.

Accurate detection of pulmonary nodules on CT is essential for correct staging and therapy optimization in children with malignancy as the lungs are one of the most common sites for metastatic disease. This task is both time-consuming and fraught with human error. Studies in adults have shown that potentially significant pulmonary nodules may be missed even by experienced radiologists [92, 93]. The development of AI algorithms, including computer-aided detection (CAD) systems, to assist radiologists in lung nodule identification has been shown to improve radiologists’ sensitivity for pulmonary nodule detection in adults when used as a second reader [94–98]. Studies have also shown decreased scan interpretation time and perceived “reduced workload for radiologists” with the use of AI assistance [99, 100]. Adult-trained CAD systems have been applied to pediatric CT data (Fig. 10). However, to our knowledge, no pediatric-specific lung nodule CAD systems have been developed using pediatric training data.

**Fig. 11:**
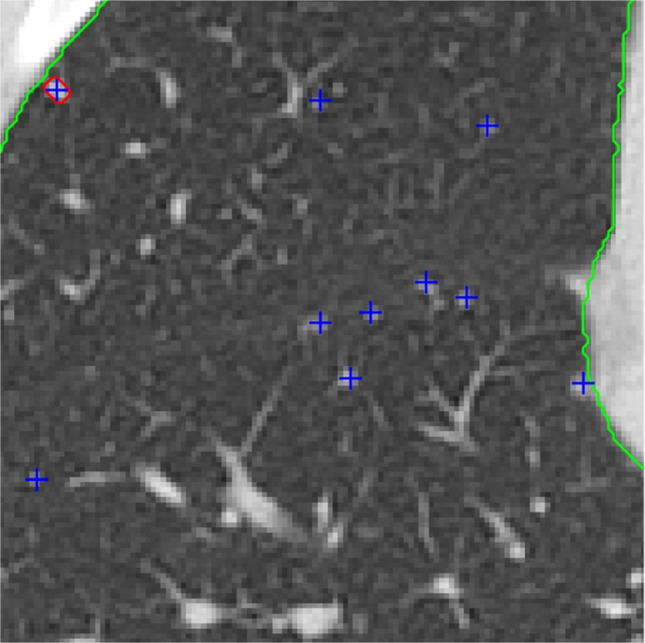
11-year-old F with follicular variant papillary thyroid carcinoma. Multiple micronodules not detected by the adult-trained CAD system are marked with blue crosshair. One CAD system detected subpleural micronodule is also present (detection with red boundary)

The application of adult-trained AI software to pediatric chest imaging for nodule detection has had mixed results. A study by Shin et al. evaluated 2273 pediatric chest radiographs using a ResNet34-based deep convolutional neural network developed and approved for adult chest radiographs for assessment of eight different pulmonary pathologies, including pulmonary nodules [101]. The AI-based software showed sensitivity, specificity, and accuracy of 93.8%, 96.6%, and 96.6%, respectively, for nodule detection. Ni et al. evaluated a deep convolutional neural network (DCNN) for the diagnosis of pulmonary nodules on chest CT in adolescent and young adult patients with osteosarcoma and found that the DCNN demonstrated significantly higher sensitivity and specificity (92.3% vs 90.8% and 55.2% vs 35.1%, respectively), and significantly greater accuracy (AUC 0.795 vs 0.687) compared with the manual detection of pulmonary nodules by junior physicians [102]. The study also found that the use of DCNN significantly reduced reading time. Hardie et al. found that two CAD systems trained on adult data had lower sensitivity for nodule detection on pediatric data (Flyer scan [103] 68.4%; Medical Open Network for Artificial Intelligence (MONAI [104]) 53.1%) than on adult data (Flyer scan 83.9%; MONAI 95.5%) [105]. Notably, Hardie et al. reported that the mean nodule size was smaller (p < 0.001) in the pediatric testing data than in the adult testing data (mean nodule size 5.4 mm vs 11 mm, respectively). Similarly, Salman et al. found that an adult-trained pulmonary nodule CAD system demonstrated lower sensitivity on pediatric chest CTs relative to two pediatric radiologists; sensitivity improved when micronodules were excluded (sensitivity 39% vs 68%) [106]. Given the variability in AI performance across these studies, and the potential advantages of improved sensitivity for nodule detection, reduced scan interpretation time, and decreased radiologist fatigue with AI detection of nodules, these findings collectively underscore the need for pediatric-specific CAD systems trained on pediatric lung imaging data (Fig. 11).

### Characterization of nodules

Nodule characterization has traditionally been limited to features detectable to the human eye, including nodule size, shape, margin, internal characteristics, and evolution over time. For example, four benign calcification patterns include diffuse, central, laminated, and popcorn calcification. Some studies suggest that nodule size greater than 5 mm in patients with underlying malignancy is suggestive of metastasis [16, 107]. Likewise, nodule growth is suspicious for malignancy. While ground glass and part solid nodules are viewed as suspicious for lung cancer in adults, given the rarity of primary pulmonary malignancy in children, ground glass nodules are more likely to represent infection/inflammation.

The use of AI has enabled further characterization of pulmonary nodules using hand-crafted radiomic and deep learning (DL) approaches. Radiomics aims to extract and analyze predefined and handcrafted quantitative features of an imaged lesion to assess its intra-tumoral properties that are invisible to the human eye, allowing for tasks such as tumor characterization and outcome prediction [108, 109]. For example, a study by Cho et al. of pulmonary nodules in children with osteosarcoma found that metastases could be differentiated from benign nodules by using computerized texture analysis, with features including higher mean attenuation (potentially reflecting ossification/calcification), greater inhomogeneity, and larger diameter being significant predictors of metastatic nodules [27]. In contrast to radiomics, DL features are not predefined and instead a neural network is trained using a large dataset to discover features that can be used to predict outcomes, which can be done supervised, semi-supervised, or unsupervised [110]. Several adult studies have evaluated the use of radiomics and deep learning for predicting lung nodule malignancy and have demonstrated excellent performance in malignancy risk stratification, with some models even extending to histologic prediction [109, 111]. An active grand challenge in biomedical imaging competition, Lung Nodule Analysis Malignancy Risk Estimation 2025, is further advancing this field [112]. By contrast, to date there has been limited application of AI techniques for nodule characterization in children. Potential benefits of improved prediction of nodule risk of malignancy include decreased false-positive findings, decreased unnecessary follow-up, patient anxiety, and health care costs.

### Future directions

The current shortage of pediatric radiologists, compounded by rising imaging volumes and the increasing complexity of examinations, poses significant challenges to maintaining timely and accurate interpretation of exams. These pressures create an opportunity for AI-based tools to improve workflow efficiency and reduce cognitive burden, which will ultimately support high-quality interpretation of pediatric imaging. Given the promise of AI algorithms for improved nodule detection and characterization in adult populations, and prior studies demonstrating inferior performance of adult-trained CAD systems when applied to pediatric CT scans, the need for pediatric-trained AI algorithms is clear. However, a significant barrier to the development of pediatric-specific lung nodule CAD systems is the current lack of publicly available annotated pediatric training and testing data. To date, there are no pediatric pulmonary nodule datasets available on The Cancer Imaging Archive [113], one of the largest public cancer imaging databases. The Children’s Oncology Group has a central image registry Quality Assurance Review Center (QARC) with datasets that include imaging from multiple different cancer types. Access to these heterogeneous, unlabeled datasets requires application to the organization. The creation of a large, labeled, and publicly available pediatric pulmonary nodule database will greatly aid in the development of pediatric-specific pulmonary nodule CAD systems targeting clinically significant nodules in children, including micronodules. In addition to the development of CAD systems for the detection of nodules on pediatric chest CT, there is also great opportunity for the creation of algorithms dedicated to the characterization of such nodules for prognostication purposes. These activities are in direct support of the Image IntelliGently™ Advocacy Campaign of the American College of Radiology that endorses the principle that “all pediatric patients will have access to clinically useful AI in radiology.”

## Conclusions

Pulmonary nodules are common in both healthy children and children with cancer. Benign etiologies are far more common than malignant ones, and metastases are far more common than primary lung malignancies. While some imaging characteristics are suggestive of metastatic disease, there are currently no definitive features of nodules that can reliably distinguish malignant from benign nodules in children. AI for nodule detection and characterization demonstrates great promise in adult patients, whereas little research has focused on pediatric pulmonary nodules. Furthermore, the few studies that have investigated the performance of adult-trained AI algorithms on pediatric data demonstrate mixed performance, revealing the need for pediatric-specific AI. Key to the development of trustable AI systems for pediatric lung nodule detection and characterization are accurately annotated pediatric datasets, which currently do not exist. Collaborative efforts across healthcare institutions, radiologists, computer scientists and engineers, and vendors are needed for the continued development of much-needed pediatric-specific AI systems.

## Data Availability

No datasets were generated or analysed during the current study.
